# Performance Under Stress: An Eye-Tracking Investigation of the Iowa Gambling Task (IGT)

**DOI:** 10.3389/fnbeh.2018.00217

**Published:** 2018-09-24

**Authors:** Boban Simonovic, Edward J. N. Stupple, Maggie Gale, David Sheffield

**Affiliations:** ^1^University of Derby Online Learning, University of Derby, Derby, United Kingdom; ^2^Department of Human Sciences, University of Derby, Derby, United Kingdom

**Keywords:** decision making, cognitive reflection test (CRT), stress, Iowa Gambling Task (IGT), somatic marker hypothesis, eye-tracking, conscious knowledge

## Abstract

Stress pervades everyday life and impedes risky decision making. The following experiment is the first to examine effects of stress on risky decision making in the Iowa Gambling Task (IGT), while measuring inspection time and conscious awareness of deck contingencies. This was original as it allowed a fine grained rigorous analysis of the way that stress impedes awareness of, and attention to maladaptive financial choices. The extended Cognitive Reflection Task (CRT) further afforded examination of the impact of impaired reflective thinking on risky decision making. Stressed participants were slower to avoid the disadvantageous decks and performed worse overall. They inspected disadvantageous decks for longer than the control condition and were slower in developing awareness of their poor deck quality compared to the control condition. Conversely, in the control condition greater inspection times for advantageous decks were observed earlier in the task, and better awareness of the deck contingencies was shown as early as the second block of trials than the stress condition. Path analysis suggested that stress reduced IGT performance by impeding reflective thinking and conscious awareness. Explicit cognitive processes, moreover, were important during the preliminary phase of IGT performance—a finding that has significant implications for the use of the IGT as a clinical diagnostic tool. It was concluded that stress impedes reflective thinking, attentional disengagement from poorer decks, and the development of conscious knowledge about choice quality that interferes with performance on the IGT. These data demonstrate that stress impairs risky decision making performance, by impeding attention to, and awareness of task characteristics in risky decision making.

## Introduction

Stress is a mental tension that arises in uncontrollable situations and results in a compensatory psychological and physiological response (Lovallo, 2016). Stress moreover, alters cognitive and emotional processes implicated in decision making (Reimann and Bechara, 2010; Simonovic et al., 2017a; Starcke et al., 2017). Traditionally, emotion was characterized as disruptive to cognitive processes in decision making however, the Somatic Marker Hypothesis (SMH) made a compelling case that emotional factors and arousal facilitate effective decision making (Damasio, 1994). The SMH postulates that emotion plays a pivotal role in complex decision making (Bechara and Damasio, 2005) and emotion-based learning (Damasio, 1996; Starcke et al., 2017). The goals of this article are two fold: first, to examine the effect of stress on cognitive reflection, conscious awareness and attention in predicting risky decision making performance; and second, to test theoretical explanations of the Iowa Gambling Task (IGT) and consider its suitability as a diagnostic clinical tool.

The IGT was developed to test the SMH and emotion-based learning, by mimicking real life decision making with risks, rewards and punishments (Bechara et al., 1994). IGT participants select cards from four decks which have different frequencies of reward and punishment. Advantageous decks offer moderate rewards and small punishments whereas disadvantageous decks offer larger rewards and substantial punishments, resulting in an overall loss. Participants should make as much notional money as possible over the course of the task. It is assumed that participants develop a “gut feeling” (or somatic marker) about the “goodness” or “badness” of decks and progressively acquire conceptual knowledge and awareness about task contingencies. Early evidence indicated that somatic markers help advantageous decision making during IGT performance (e.g., Bechara et al., 1994; Bechara and Damasio, 2005), however subsequent studies challenged this view—showing that analytic thinking and explicit knowledge of the deck contingencies played a more significant role in the IGT than previously thought (e.g., Maia and McClelland, 2004; Bowman et al., 2005; Simonovic et al., 2017a,b). Indeed, Simonovic et al. (2017a,b) argued that cognitive processes and conscious awareness influence the development of somatic markers and suggested that the IGT performance is best understood through the interplay between emotional and analytic processes within a dual process account (see e.g., Kahneman, 2011).

Brevers et al. (2013) use a dual-process framework that contrasts intuitive, effortless, emotional and unconscious processes (Type I) with effortful conscious and controlled processes (Type II). The SMH can account for intuitive processes such as emotional responses or gut feelings that shape IGT performance and are measured through physiological techniques (Glöckner and Witteman, 2010). Brevers et al. (2013) further proposed that “cool” reflective processing is needed for evaluation of “hot,” affective choices made early in the IGT. Hence, current evidence suggests a complex interplay between Type I and Type II processes in determining IGT performance. These Type II processes require further investigation by measuring attention and conscious awareness about the task.

### Stress

According to the SMH, the connection between somatic markers and decision making can be interrupted by stress (e.g., Reimann and Bechara, 2010). Stress interferes with the learning process in healthy controls, increases risk-taking behavior and leads to disadvantageous card selections on the IGT (Preston et al., 2007; Miu et al., 2008; van den Bos et al., 2009; Robinson et al., 2015; Simonovic et al., 2017a; Starcke et al., 2017). Preston et al. (2007) demonstrated that stressed participants learned more slowly on the IGT than the control group. Thus, incidental anticipatory stress interfered with the development of somatic markers which may have been due to working memory disruption (Hinson et al., 2002). Furthermore, Preston et al. (2007) suggested that anticipatory stress shifts cognitive processes away from deliberative processing towards automatic processing, and thus impairs differentiation between advantageous and disadvantageous choices—a proposal that warranted further investigation.

There is however disagreement about the nature of the impact of stress on learning and performance in the IGT. Starcke et al. (2017) argued that performance on the early trials of the IGT is determined by emotional feedback processing. However, when Simonovic et al. (2017a) replicated and extended Preston et al. (2007) work, demonstrating that reflective thinking was also important early in the task. The correlations reported in Simonovic et al.’s (2017a) study also indicated that reduced reflective thinking in the stress group led to increased disadvantageous deck selection. The effect of stress on IGT performance was predicted by analytic thinking and thus challenged the primacy of emotional learning in early trials (see Bechara et al., 2000). The results from these studies resonate with previous research suggesting that stress reduces cognitive capacity and consequently diminishes learning from negative choices (Lighthall et al., 2009; Petzold et al., 2010). However, it is difficult to unpack whether impaired IGT performance is due to stress inhibiting the development of somatic markers or the reduced capacity for reflective thinking, or both.

Stress disrupts cognitive control (Schwabe and Wolf, 2009), and changes from goal-directed to automatic control of action (Margittai et al., 2015). Margittai et al. (2015) tested participants who received placebo, cortisol and yohimbine, a drug that increases noradrenergic stimulation, before performing the Cognitive Reflection Task (CRT; Frederick, 2005). Their results showed that an increase in cortisol reduced reflective processing and increased intuitive processing. The results showed that cortisol mediates the engagement of cognitive processes and supports the view that stress during the IGT reduced capacity for Type II processes (e.g., Simonovic et al., 2017a). Margittai et al.’s (2015) results also accord with research showing Type II processes to be cortisol dependent (Schwabe and Wolf, 2009, 2013).

Conscious awareness choice quality is important in risky decision making generally, and also the IGT. Maia and McClelland (2004) challenged Bechara et al.’s (1994) view that conscious awareness and performance on the IGT are unrelated by showing that explicit knowledge about decks improved deck selection. Bowman et al. (2005) further demonstrated an explicit evaluation of affective choices could guide future decision making. Fernie and Tunney (2013) however, showed that not all participants attain conscious awareness of deck quality, and that conceptual knowledge was not essential for advantageous selections. Newell and Shanks (2014) further argue that conscious awareness diverts attention to positive choices and recruits Type II goal-directed cognitive processes. Thus, conscious awareness initiates executive attention that further prompts executive functioning and enhances decision making. Thus, risky decision making can be influenced by both emotion-mediated, explicit knowledge and analytic thought, but the precise nature of the interaction between these processes remains an open question, particularly under stressful conditions.

While the impact of stress on conscious awareness during the IGT has not been investigated there is evidence that stress impedes attentional monitoring of the accuracy of choices (Reyes et al., 2015) and impedes information processing (Hardy et al., 1996). Furthermore, stress may impair preconscious selective attention to avoid negative stimuli (e.g., Roelofs et al., 2007). These findings have implications for explaining the way that stress impairs IGT performance and risky choice *per se*.

The present study extends Simonovic et al. (2017a) using an eye-tracking methodology; an extended, more reliable CRT (Toplak et al., 2014, see Stupple et al., 2017) and Maia and McClelland’s (2004) Conscious Awareness Test to provide a more comprehensive analysis of when and where Type II processes are implicated during the IGT. These analyses are informative for testing theories of IGT performance, but also understanding risky decision making under stress.

We propose that stress impairs decision making by reducing capacity for analytic processing which impedes attention to, and awareness of, situational factors. Thus, we propose that when performing IGT under stress, analytic processing would be less predictive of performance; that participants will persist in fixating on disadvantageous decks for more trials and show less awareness of task characteristics.

This leads to a series of hypotheses about: (1) performance; (2) the role of reflection; (3) inspection times; (4) conscious awareness; and (5) predicting IGT performance. Thus: (1) the stress manipulation will inhibit performance on the IGT and delay the elimination of disadvantageous deck selections; (2) there will be more significant correlations between CRT and IGT performance in the control condition than the stress conditions; (3) there will be increased inspection time for disadvantageous decks which will persist across more blocks in the stress condition. There will also be decreased inspection time for advantageous decks which will persist across more blocks in the stress condition; (4) there will be poorer estimates of deck quality in the stress condition; and finally, (5) the relationship between inspection time, conscious knowledge, CRT scores, stress and IGT performance will be tested by path analyses; we predict that inspection time, conscious knowledge, CRT scores and Systolic Blood Pressure (SBP) reactivity will indirectly predict IGT performance.

## Materials and Methods

### Participants

Twenty-three male and 53 female undergraduate students aged 19–56 years, were recruited and randomly allocated to stress and control groups. Participants had normal or corrected to normal vision. This study was carried out in accordance with the recommendations of the British Psychological Society. The protocol was approved by the University of Derby Human Sciences Research Ethics Committee. All subjects gave written informed consent in accordance with the Declaration of Helsinki. People under the age of 18 years old and people who reported depression, anxiety, any cardiovascular disease, high blood pressure or a history of neurological illnesses were excluded from participation.

### Materials

#### Stress Manipulation

The study used an anticipatory speech task (Simonovic et al., 2017a), based on a modified version of Preston et al. (2007) anticipatory speech task. A video camera was installed that simulated recording; before the experiment; only participants in the experimental group were told that they would be video-recorded during their performance and they would have to deliver a speech to summarize their experience at the end of the experiment. Control participants were not exposed to any aspect of the stress manipulation while completing the experiment.

#### Physiological Measurement

SBP and Heart Rate (HR) responses to stress were measured to check whether the manipulation was effective using a continuous, non-invasive cardiovascular Finometer (Finapres Medical System, Amsterdam, Netherlands). Baseline SBP and HR measurements were taken for 5 min before the initiation of IGT followed by SBP and HR measurements taken during the IGT performance. SBP and HR reactivity were calculated by subtracting the average of the performance SBP/HR measurements from the average of resting SBP/HR measurements.

#### Conscious Awareness Test

Maia and McClelland’s (2004) Test of Awareness measures the emergence of conceptual knowledge about deck contingencies. We obtained deck ratings of −10 to +10 to measure awareness of deck quality (Deck Rating).

#### Eye Tracking Measurements

Eye movements were recorded with the Eye-gaze binocular system Tobii-X2-30 (Inquisit 4 ms plugins), with a remote binocular sampling rate of 30 Hz and an accuracy of approximately 0.45°. The X2 Eye Tracker is a stand-alone eye tracker, and was attached to a laptop (Dell, Precision M6700, 2.70G hz). Participants were seated approximately 0.7 m from the laptop monitor. The Tobii measured 184 mm in length and enabled tracking at close distances (up to 36° gaze angle). Fixations were identified using a fixation radius of 20 pixels and a minimum fixation duration of 100 ms or above. Before starting the experiment, a 9-point calibration routine was executed. Each data point was identified with a timestamp and “X, Y” coordinates, and these coordinates were processed further into fixations and overlaid on a video recording of the IGT. Choices, decision times, and basic eye-tracking parameters such as inspection time and coordinates were recorded. To avoid methodological artifacts, eye tracking metrics were delineated through fixation filters. Eye-tracking parameters such as were recorded for both eyes and then aggregated. To avoid methodological artifacts, eye tracking metrics were delineated through fixation filters. Fixation filters were used to remove blinking points and extrapolate the data correctly into fixations. Non-overlapping areas of interest (AOI) around each cell in the matrix were defined, each containing different decks. Hence, four AOIs were obtained with the size of 690 × 458 pixels for decks (36° gaze angle). For each participant and each decision, the inspection time within each AOI was calculated.

#### IGT

Bechara et al.’s (1994) computerized version of IGT and standard instructions were used. Inquisit 4 (Millisecond Software; Seattle, WA, USA) was used to run the IGT script; participants were required to choose individual cards from four decks that provide financial rewards and punishments. Bechara et al.’s (1994) IGT instructions for computerized version were followed. One-hundred and forty trials (seven Blocks of 20) were completed.

#### CRT

The seven-item CRT (Toplak et al., 2014) was used to measure analytic ability. The score was the total number of correct answers. Higher CRT scores indicated higher reflective ability. The CRT consists of problems where an intuitive answer must be resisted to reach the correct solution. An example question is “If John can drink one barrel of water in 6 days, and Mary can drink one barrel of water in 12 days, how long would it take them to drink one barrel of water together?” The correct answer is 4 days and the intuitive answer is 9 days. The Cronbach alpha for correct CRT responses was α = 0.77.

#### Procedure

Following consent, participants sat for a 5-min resting period, and then baseline SBP/HR measurements were taken. Next, they were randomly allocated to groups; they were no differences in age or gender. The instructions regarding the presentation to the camera were only given to the experimental group, and they were shown the camera which was then switched on. The CRT was administrated followed by the IGT. Eye tracking measures and SBP/HR measurements were taken continuously during the IGT performance. Also, conscious awareness per Block was assessed during the task. After the completion of the IGT task, participants in the experimental group were told that they would not have to give the speech at that point. Finally, participants were debriefed, and post-task SBP/HR measurements were taken to ensure readings had returned to baseline.

#### Analytic Strategy and Scoring

Initial analyses focused on checking that the stress manipulation was effective: ANOVA was used to determine if SBP and HR reactivity differed by condition. Next, a 2 (condition) × 7 (Block) mixed ANOVAs were used to determine the effect of the manipulation on IGT scores across the seven Blocks. Standard scoring was derived by deducting total disadvantageous card picks (A + B) from total advantageous picks (C + D).

As parametric assumptions were not met, a Mann–Whitney test was used to test for differences in CRT scores between the two conditions. Bivariate correlations were examined relationships between CRT scores and disadvantageous deck picks (A + B) during each Block, for each group separately.

Inspection time was also examined across Blocks; 2 (condition) × 7 (Block) mixed ANOVAs were used to determine the differences in inspection time for disadvantageous and advantageous decks across Blocks. Next, a 2 (condition) × 7 (Block) mixed ANOVA was used to determine the effect of manipulation on overall deck ratings (C + D – A + B) across Blocks.

**Figure 1:**
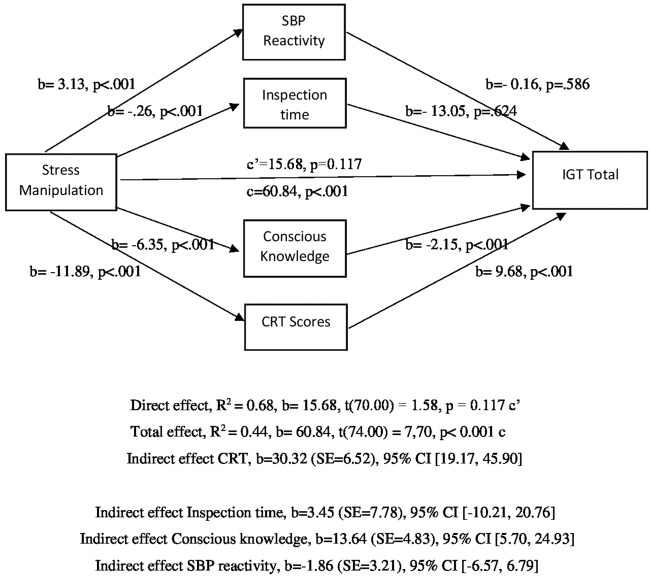
Model of stress manipulation as a predictor of Iowa Gambling Task (IGT) scores, mediated by Systolic Blood Pressure (SBP) reactivity, inspection time, Cognitive Reflection Task (CRT) and conscious knowledge. The Confidence Interval (CI) for the indirect effect is a BCa bootstrapped CI based on 10,000 samples.

Finally, a bootstrapped mediation model tested the conceptual model outlined in Figure 1. All hypotheses were tested simultaneously using the Process macro for SPSS (Hayes, 2012), with 10,000 bootstrapping re-samples and bias-corrected 95% Confidence Intervals (CIs) for each indirect effect. In bootstrapping analyses, bias-corrected CIs that do not contain 0 signify a significant mediational effect (Preacher and Hayes, 2004, 2008). Direct effects estimate how much two cases differing on the independent variable (stress manipulation) also differ on the dependent variable (total IGT score: (C + D) − (A + B)), independent of the effect of the mediator variables (SBP reactivity, inspection time, CRT scores and conscious knowledge) on the dependent variable. Total effects are the sum of the indirect and direct effects of the independent variable (stress manipulation) on the dependent variable (IGT scores; Hayes, 2012). To balance concerns related to Type I and Type II errors the alpha level for all analyses was adjusted to *p* < 0.005. Analysis was conducted using IBM SPSS 24 for Windows. All analyses were repeated with gender as either a covariate or a moderator; no outcomes were affected.

## Results

### Manipulation Checks

*Stress induction—*ANOVA revealed a condition (stress vs. control) effect for SBP reactivity, *F*_(1,74)_ = 13.63, *p* < 0.001, $\eta_{\text{p}}^{2}$ = 0.16; reactivity was larger in the stress condition than in the control condition (Table 1). Further, ANOVA revealed a condition (stress vs. control) effect for HR reactivity, *F*_(1,74)_ = 4.07, *p* < 0.05, $\eta_{\text{p}}^{2}$ = 0.05; reactivity was larger in the stress condition than in the control condition (Table 1).

**Table 1:** Mean (SD) Systolic Blood Pressure (SBP) and Heart Rate (HR) at baseline and during Iowa Gambling Task (IGT) performance.

	SBP		HR	
	Baseline	During	Baseline	During
Stress	122.05 (15.19)	139.16 (13.76)	80.46 (14.44)	85.08 (14.28)
Control	120.22 (8.70)	122.60 (7.19)	77.33 (7.95)	77.50 (8.24)

Cognitive reflection task (CRT) performance under stress— A Mann–Whitney test showed differences in CRT scores between the two groups: participants in the stress condition had lower CRT scores (Median = 1, IQR = 5) than participants in the control condition (Median = 4.5, IQR = 7) *U* = 128, *p* < 0.001, *r* = 0.72 demonstrating that reflective thinking was inhibited by stress.

### IGT Performance—Deck Selection Analysis

A Greenhouse-Geisser adjusted ANOVA was used to determine the effect of stress condition on the standard IGT scoring, (C + D) − (A + B) across the seven Blocks of the IGT. There was a main effect of stress condition *F*_(1,74)_ = 58.80, *p* < 0.001, $\eta_{\text{p}}^{2}$ = 0.44, participants in the stress condition had lower IGT scores than the control group. There was a main effect of Block, *F*_(4.25,314.32)_ = 33.29, *p* < 0.001, $\eta_{\text{p}}^{2}$ = 0.31. with IGT scores increasing after the first Block. The adjusted *post hoc* pairwise comparisons demonstrated that IGT scores in Block 1 were significantly lower than all other Blocks (all *p* < 0.005). Furthermore, IGT scores in Block 2 were significantly lower than Blocks 4, 5, 6 and 7 (all *p* < 0.005). There were no other significant differences between Blocks. There was a significant Block × Stress manipulation interaction, *F*_(4.25,314.32)_ = 7.44, *p* < 0.001, $\eta_{\text{p}}^{2}$ = 0.09, independent *t*-tests revealed that participants in the stress conditions had lower IGT scores in all but Block 1 (all significant *p* < 0.001) compared with participants in the control condition (see Table 2 for descriptive statistics).

**Table 2:** Mean (SD) standard IGT scores per Block for control and stress group.

Blocks	Stress	Control	Total
1	−4.05 (4.48)	−3.16 (8.29)	−3.60 (6.63)
2	−2.32 (6.39)*	4.53 (8.02)	1.10 (7.98)
3	−0.53 (7.56)*	9.40 (6.94)	4.43 (8.77)
4	−0.89 (6.23)*	10.32 (7.22)	4.71 (8.76)
5	2.00 (7.45)*	11.74 (7.70)	6.87 (8.99)
6	1.21 (7.81)*	12.10 (7.88)	6.65 (9.53)
7	1.89 (9.21)*	13.16 (7.70)	7.53 (10.16)
Total	−2.68 (31.15)	58.16 (37.47)	

*Denotes significant p < 0.005.

### Correlations Between CRT and IGT by Block

Correlations between disadvantageous card selections (A + B) per Block revealed medium to large correlations across both conditions. Further correlations between disadvantageous card selection scores for each Block and CRT scores were calculated for control and stress conditions separately. Significant negative correlations between disadvantageous card selection scores and CRT scores were observed in Blocks 1, 6 and 7 in the stress condition, and in Blocks 3, 5, 6 and 7 in the control condition. Higher CRT scores were associated with better performance in those Blocks (Table 3).

**Table 3:** Correlations between CRT scores and disadvantageous card selection scores (A + B).

Blocks	Stress	Control
1	*r* = −0.321, *p* = 0.049	*r* = 0.007, *p* = 0.966
2	*r* = −0.005, *p* = 0.977	*r* = −0.186, *p* = 0.264
3	*r* = −0.175, *p* = 0.294	*r* = −0.442, *p* = 0.005*
4	*r* = −0.124, *p* = 0.458	*r* = −0.201, *p* = 0.227
5	*r* = −0.234, *p* = 0.158	*r* = −0.476, *p* = 0.003*
6	*r* = −0.354, *p* = 0.029	*r* = −0.519, *p* = 0.001*
7	*r* = −0.327, *p* = 0.045	*r* = −0.484, *p* = 0.002*
Total	*r* = −0.354, *p* = 0.029	*r* = −0.462, *p* = 0.003*

*Denotes significant p < 0.005.

### Deck Inspection-Time Analyses

A Greenhouse-Geisser-adjusted ANOVA with log transformed data was used to determine the effect of stress condition on the inspection time for disadvantageous choices (A + B) across the seven Blocks of the IGT. There was: a main effect of stress condition, *F*_(1,74)_ = 89.25, *p* < 0.001, $\eta_{\text{p}}^{2}$ = 0.54, such that longer inspection-times were observed in the stress group compared to the control group. There was a main effect of Block, *F*_(4.14,306.16)_ = 21.81, *p* < 0.001, $\eta_{\text{p}}^{2}$ = 0.23, such that there was an increase in inspection time from Block 1 until Block 4 (all *p* < 0.001). The *post hoc* pairwise comparisons (threshold alpha *p* < 0.005) demonstrated that inspection time in Block 1 was lower than inspection time in Blocks 2 and 3 (all *p* < 0.005). Inspection times in Blocks 2 and 3 were significantly higher than inspection times in Blocks 6 and 7. Furthermore, inspection time in Block 4 was significantly higher than inspection times in Blocks 5, 6 and 7 (all *p* < 0.005). There was a significant Block × stress manipulation interaction, *F*_(4.14,306.16)_ = 26.16, *p* < 0.001, $\eta_{\text{p}}^{2}$ = 0.26. Independent *t*-tests revealed longer inspection time in the stress conditions (*p* < 0.005), in all but Block 1, 6 and 7 compared with participants in the control condition (see Table 4 for descriptive statistics).

**Table 4:** Mean (SD) inspection time for disadvantageous decks per Block for control and stress group.

Blocks	Stress	Control	Total
1	0.21 (0.08)	0.23 (0.15)	0.22 (0.12)
2	0.45 (0.14)*	0.15 (0.14)	0.30 (0.20)
3	0.49 (0.18)*	0.13 (0.08)	0.31 (0.23)
4	0.53 (0.20)*	0.17 (0.17)	0.35 (0.26)
5	0.38 (0.23)*	0.12 (0.10)	0.25 (0.22)
6	0.20 (0.16)	0.13 (0.12)	0.17 (0.15)
7	0.19 (0.15)	0.14 (0.14)	0.17 (0.15)
Total	0.35 (0.10)	0.15 (0.07)	

*Denotes significant p < 0.005.

A Greenhouse-Geisser-adjusted ANOVA with log-transformed data was used to determine the effect of stress condition on the inspection time for advantageous choices (C + D) across the seven Blocks of the IGT. There was: a main effect of stress condition *F*_(1,74)_ = 7.52, *p* = 0.008, $\eta_{\text{p}}^{2}$ = 0.09, such that longer inspection time was observed in the control group compared to the stress group. There was a main effect of Block, *F*_(3.78,280.19)_ = 12.12, *p* < 0.001, $\eta_{\text{p}}^{2}$ = 0.14, such that there was an increase in inspection time from Block 1 until Block 4 (all *p* < 0.05). The *post hoc* pairwise comparisons (*p* < 0.005), demonstrated that inspection time for advantageous decks in Block 1 was significantly lower than Blocks 3 and 4 (all *p* < 0.005). Furthermore, inspection time for advantageous decks in Blocks 3 was significantly higher than Blocks 5 and 6 (all *p* < 0.005). Additionally, inspection time for advantageous decks in Block 4 was significantly higher than Blocks 5, 6 and 7 (all *p* < 0.005). There was a significant Block × stress manipulation interaction, *F*_(3.78,280.19)_ = 10.88, *p* < 0.001, $\eta_{\text{p}}^{2}$ = 0.13, on inspection time. Independent *t*-tests revealed longer inspection time for participants in the control condition in Blocks 2, 3 and 4 compared with participants in the stress condition (see Table 5 for descriptive statistics).

**Table 5:** Mean (SD) inspection time for advantageous decks per Block for control and stress group.

Blocks	Stress	Control	Total
1	0.22 (0.10)	0.22 (0.14)	0.22 (0.12)
2	0.19 (0.10)*	0.30 (0.14)	0.25 (0.13)
3	0.23 (0.15)*	0.41 (0.23)	0.32 (0.21)
4	0.23 (0.16)*	0.46 (0.30)	0.35 (0.27)
5	0.19 (0.12)	0.22 (0.18)	0.21 (0.15)
6	0.23 (0.15)	0.18 (0.16)	0.21 (0.16)
7	0.23 (0.14)	0.19 (0.18)	0.21 (0.17)
Total	0.22 (0.08)	0.29 (0.10)	

*Denotes significant p < 0.005.

### Conscious Awareness of Deck Quality

A Greenhouse-Geisser-adjusted ANOVA was used to determine the effect of stress condition on the overall deck ratings (C + D − A + B) across the seven Blocks of the IGT. There was: a main effect of stress condition *F*_(1,73)_ = 12.90, *p* = 0.001, $\eta_{\text{p}}^{2}$ = 0.15, such that the stress deck ratings were lower compared to the control group. There was a significant main effect of Block, *F*_(2.75,200.67)_ = 26.43, *p* < 0.001, $\eta_{\text{p}}^{2}$ = 0.27, such that there was increase in deck ratings across blocks. The adjusted (*p* < 0.005) *post hoc* pairwise comparisons demonstrated that deck ratings in Block 1 was significantly lower than all Blocks (all *p* < 0.001). There were no other significant differences between Blocks. and a non-significant Block × stress manipulation interaction, *F*_(2.75,200.67)_ = 1.62, *p* = 0.13, $\eta_{\text{p}}^{2}$ = 0.02, on overall deck ratings. This indicated that participants in the control conditions started to develop understanding of the patterns of gains and losses after the first Block (see Table 6 for descriptive statistics).

**Table 6:** Mean (SD) for overall deck ratings (C + D − A + B) per Block for control and stress group.

Blocks	Stress	Control	Total
1	−5.84 (7.48)	1.37 (7.01)	−2.28 (8.07)
2	1.18 (9.36)	4.83 (9.62)	2.99 (9.13)
3	1.60 (8.64)	8.37 (8.81)	4.94 (9.31)
4	3.47 (9.28)	8.51 (11.15)	5.96 (10.49)
5	3.13 (8.08)	9.51 (11.38)	6.28 (10.30)
6	2.39 (8.27)	10.48 (10.65)	6.39 (10.30)
7	2.45 (8.00)	10.16 (10.46)	6.25 (10.01)
Total	1.19 (6.90)	7.61 (8.32)	

### Path Analyses of Determinants of IGT Performance

The assumption of normality was met and there were no outliers. For the path analyses, stress manipulation was an independent variable and overall IGT score was the dependent variable. CRT scores, inspection time, conscious knowledge and SBP reactivity were indirect pathways. Initially, it was checked if the stress manipulation predicts chosen mediators. Stress manipulation significantly predicted all the mediators (Table 7).

**Table 7:** The overall model and effect of stress manipulation on mediators.

	Overall model	Stress manipulation effect
CRT	*F*_(1,74)_ = 80.35, *p* < 0.001, *R*^2^ = 0.52	*b* = 3.13 (SE = 0.35), *t*_(74)_ = 8.96, *p* < 0.001
SBP reactivity	*F*_(1,74)_ = 21.54, *p* < 0.001, *R*^2^ = 0.22	*b* = −11.89 (SE = 2.56), *t*_(74)_ = −4.64, *p* < 0.001
Inspection time	*F*_(1,74)_ = 90.93, *p* < 0.001, *R*^2^ = 0.55	*b* = −0.26 (SE = 0.03), *t*_(74)_ = −9.54, *p* < 0.001
Conscious knowledge	*F*_(1,74)_ = 12.98, *p* < 0.001, *R*^2^ = 0.15	*b* = −6.35 (SE = 1.76), *t*_(74)_ = −3.60, *p* < 0.001

The results were significant for all the indirect pathways. Further path analyses indicated that the direct effect of stress manipulation on IGT was not significant when controlling for indirect pathways, *b* = 15.30 (SE = 12.15), *t* = 1.26, *p* = 0.21. However, there was a significant indirect effect of stress manipulation on IGT scores through CRT, *b* = 30.32 (SE = 7.40), *Z* = 4.09, *p* < 0.001 and conscious knowledge, *b* = 13.64 (SE = 4.67), *Z* = 2.92, *p* = 0.003. Conversely, the indirect effect of stress manipulation through inspection time, *b* = 3.45 (SE = 7.07), *Z* = 0.49, *p* = 0.63, and SBP reactivity, *b* = −1.86 (SE = 3.50), *Z* = −0.53, *p* = 0.49 was not significant. The full model of stress manipulation as a predictor of IGT scores is outlined in Figure 1.

## Discussion

To summarize our findings: compared to the control condition, stressed participants were slower to avoid the disadvantageous decks and performed worse overall, they inspected disadvantageous decks for longer, and were slower in developing awareness of their poor deck selections compared to the control condition. Conversely, the control condition had longer inspection times for advantageous decks earlier in the task, and earlier accuracy in awareness of the deck contingencies than the stress condition. Path analysis demonstrated that stress reduced performance by impeding reflective thinking and conscious awareness. We now present a more detail review of the findings in relation to our hypotheses.

Inspection time differences between disadvantageous and advantageous decks were observed in the stress condition persisted for longer than in the control group. It was hypothesized that the stress would inhibit the learning of deck contingencies. Participants in the control conditions had more accurate estimates of deck quality in all but Blocks 2 and 4. Finally, path analysis examined direct and indirect effects of the stress manipulation, SBP reactivity, inspection time, CRT and conscious knowledge upon IGT scores. This analysis demonstrated the stress manipulation indirectly affected IGT scores by reducing cognitive reflection and conscious knowledge but did not have a direct effect. These findings are discussed in turn.

The hypotheses related to replication of Simonovic et al.’s (2017a) findings were partially supported. The stress manipulation delayed the optimization of deck selections and reduced reflective ability as indexed by CRT scores. It was hypothesized that CRT scores would correlate in the earlier Blocks for both conditions—this was not consistently observed; however, CRT scores and disadvantageous deck picks were correlated in Block 3, 5, 6 and 7 in the control condition but were not significant in the stress condition.

### Manipulation Check and IGT Performance

The stress manipulation successfully increased SBP/HR reactivity. The results showed that stressed participants selected more cards from disadvantageous decks, after the first Block, indicating that their learning was impaired. These findings support Simonovic et al.’s (2017a) findings on a standard extended version of the IGT. These data support previous findings that stress impairs learning and leads to a slower elimination of disadvantageous deck selection (Preston et al., 2007; Starcke et al., 2017; Wemm and Wulfert, 2017). Our findings show that deck selection in the stress condition improved after the fourth trial compared to the control condition where deck selection improved after the first trial.

### CRT Results

As with Simonovic et al.’s (2017a), participants in the stress condition had significantly lower CRT scores, indicating that stress reduced reflective ability. These data support the dual process account of IGT where it is assumed that “cool” reflective processes are important in overriding “hot” processes that favor short-term gain (Brevers et al., 2013). According to Brevers et al. (2013) “cool” systems are associated with monitoring options associated with risk and gain. When “hot” systems do not allow risk assessments of the choices, “cool” systems evaluate the risk and benefits of the choices. The overall correlation between the CRT scores and disadvantageous deck selections observed for both conditions indicate that reflective processes are implicated in disambiguation of the disadvantageous deck contingencies. Correlational data further indicate that the reflective processing is significant in both early and late trials (e.g., Simonovic et al., 2017a) rather than being located only when participants have learned the rules of the task (e.g., Starcke et al., 2017). The CRT scores in the control condition correlated with disadvantageous deck selection from early in the task. This indicates that the importance of reflective thinking emerged after the second Block and persisted until the end of the task in the control condition, showing that less reflective participants were more likely to make a disadvantageous choice. However, reflective processing was not a reliable correlate with performance in the stress condition.

This is also in line with Margittai et al.’s (2015) study that demonstrated that higher cortisol levels impaired performance on the original CRT. This indicates that stress disrupts higher order control, mediated by Prefrontal Cortex (PFC; e.g., Arnsten, 2009; Schwabe and Wolf, 2011, 2013). Since neurochemicals released in response to stress (e.g., dopamine and glucocorticoids) have receptors in the PFC, decision making processes that depend on PFC can be directly affected by stress (e.g., Preston et al., 2007). This also supports Preston et al.’s (2007) argument that under stress, decision making shifts away from deliberative PFC processing towards subcortical areas of the brain and allows automatic, amygdala-mediated processing to dominate. This can explain non-optimal performance and a lack of reflective thinking in the stress condition. Although, cortisol was not measured in this study, the success of the stress induction technique (evidenced by blood pressure and HR responses) means that it is highly likely that cortisol levels affected learning and consequent performance.

### Inspection Time

Greater inspection time for disadvantageous decks was observed for participants in the stress condition, particularly from Blocks 2 to 5. One possibility is that there are differences in attentional control between the two conditions due to impaired ability to disengage from the negative choices associated with disadvantageous decks in the stress condition. It may be the case that these findings indicate that participants are merely looking at the decks they select, however the eye-tracking measure is a much finer-grained index of participant behavior and would pick up on subtle differences in attentional focus that a gross deck selection measure may miss. These data are consistent with findings where increased attention towards negative choices are associated with increased negative preferences and poorer learning from punishment (Sapolsky, 2000; Ononaiye et al., 2007; Sposari and Rapee, 2007; Cavanagh et al., 2011). Thus, the findings support the proposal that stress inhibited attentional disengagement from the negative choices. Alternately, the stress condition may have reduced participant learning from feedback by “hijacking” cognitive processes such that somatic markers or deck outcomes were attended to less. Greater inspection time for advantageous decks was observed in Blocks 2, 3 and 4, in the control condition before participants are expected to show awareness of the advantageousness of decks according to traditional SMH accounts (e.g., Bechara et al., 2000; Brand et al., 2007; Starcke et al., 2017). Thus, it is possible that stress impaired both learning from the positive and negative feedback of disadvantageous/advantageous decks, reduced participants’ ability for attentional disengagements of disadvantageous choices and increased an awareness of the advantageousness of the good decks in control group.

### Conscious Awareness

On average participants in the control condition showed sufficient knowledge of deck quality to guide advantageous long-term choices after the first Block. Deck rating scores in the stress condition suggest that stress interfered with learning as participants failed to develop sufficient knowledge to guide their performance compared to the control group. These data are consistent with previous studies that have shown that the IGT can be performed through access to explicit, conscious knowledge (e.g., Maia and McClelland, 2004, 2005; Fernie and Tunney, 2006, 2013). Thus, while the possibility that somatic markers contribute to IGT performance cannot be ruled out, the results reliably show that stress impaired the conscious processes which are integral to IGT performance. However, it should be noted that the nature of the IGT and the design of Maia and McClelland’s (2004) test could promote additional cognitive rather than intuitive processing, because participants are asked to assess deck quality at regular intervals. The present results are, however, in line with the Simonovic et al. (2017a) findings where no measure of conscious awareness was employed. Thus, these data challenge previous research that suggests non-conscious intuitive signals dominate decisional choices in the early stages of the IGT (e.g., Bowman et al., 2005; Maia and McClelland, 2005; Fernie and Tunney, 2006, 2013).

Differences in conceptual knowledge and inspection time for advantageous decks emerged after the second Block. This indicated that the control group had gained sufficient knowledge about the deck contingences and was more focused on the good decks. This also suggested that explicit knowledge runs in parallel with deck selection. Thus, good awareness of deck quality activates cognitive processing about the payoff structure leading to a more optimal decision making strategy. Konstantinidis and Shanks (2014) reported similar findings in a study that used wagering to examine conceptual awareness; participants developed preferences towards the advantageous decks and accurately justified their preferences. This is consistent with Newell and Shanks’s (2014) suggestion that conscious awareness diverts attention to positive decisional choices and recruits cognitive processes related to goal-directed behavior. According to this view, conscious awareness initiates executive attention that further initiates executive functioning (e.g., working memory) to reflect on the specific components of the task. Our data indicated that conscious awareness, attentional processing and analytic ability arise early in the task for the control group, however the precise nature of the correlates and causal relationships with learning and performance require further investigation. The results indicated that participants who were more reflective have greater awareness of deck contingences and are more focussed on good decks earlier in the task, implicating Type II processing throughout the task.

### Path Analysis

Path analysis revealed that the effect of stress on performance occurs through reduced reflective and conscious awareness rather than different routes. Furthermore, knowledge of deck quality emerged as an additional mediator to reflective ability. The mediators reduced the direct effect of stress manipulation on IGT scores. However, the analysis revealed a weak relationship between the mediators and overall IGT scores. This raises the possibility that the mediators are not strongly related to each other. Preston et al. (2007) and Simonovic et al. (2017a) argued that stress disrupts Type II cognitive processes indicating that performance was not primarily dependent on emotional processing and is more consistent with Brevers et al. (2013) dual process account of the IGT. However, contrary to Brevers et al.’s (2013) suggestion that “hot,” Type I processes guide successful performance, our data indicate that Type II processes guide decision making in the absence of stress. Emotional processing may guide Type II processing early in the task, as it could be argued that a complex interaction between these components give rise to somatic markers. Unpacking this issue is complicated by the evidence that conflict between Type I and Type II processes can be physiologically arousing (e.g., Evans, 2003; De Neys and Glumicic, 2008; De Neys et al., 2010). Thus, it could be argued that rather than emotional processing generating the arousal, it is instead due to the cognitive effort being employed to learn deck contingences.

## Conclusion

In conclusion, this experiment provides the first examination of conscious awareness under stress on the IGT and the first direct measure of attentional focus during IGT performance. The results of this experiment further clarify the findings from studies that demonstrate a link between stress and IGT performance. Moreover, we demonstrated the importance of reflective cognition, attention and conscious knowledge in later trials but also in the earlier trials traditionally associated with learning rather than performing the task. Induced stress not only impaired decision making performance, but also impeded attention to, and awareness of task characteristics in risky decision making. This was evidenced with the reduced capability for Type II thinking under stress and increased dominance for Type I thinking. These findings are also problematic for the use of the IGT as a diagnostic clinical tool—the importance of reflective processing, early conscious awareness of deck quality and impairment of these through stress all undermine the view that the IGT can diagnose a specific impairment of emotional processes. The results of this experiment support a dual-process account of risky decision making as conscious and effortful processing is impaired in the presence of stress and implicated in its absence.

## Author Contributions

All authors (BS, ES, MG and DS) made substantial contributions to the conception and design of the study and revised the manuscript for important intellectual content. BS collected the data and drafted the initial manuscript. BS, ES and DS analyzed and interpreted the data.

## Conflict of Interest Statement

The authors declare that the research was conducted in the absence of any commercial or financial relationships that could be construed as a potential conflict of interest.
